# Supplementary material for: Phylogenomics-guided discovery of a novel conserved cassette of short linear motifs in BubR1 essential for the spindle checkpoint
Source: Open Biol. 2016 Dec 21;6(12):160315. doi: 10.1098/rsob.160315 (PMC5204127; doi:10.1098/rsob.160315)
Supplement: Supplementary procedures and discussion [file rsob160315supp1.docx]

**Supplementary Information**

**Supplementary sequence file 1 - Full-length sequences of MadBub orthologs used in this study -** fasta file containing full-length sequences of MadBub gene family specifically selected for this study. Headers include a four-letter species code (see for ID conversion **supplementary table I**) followed by MADBUB, BUB or MAD to indicate the ancestor and the two subfunctionalized paralogs, respectively (e.g. >HSAP_BUB or DDIS_MADBUB).

**Supplementary sequence file 2 – Conserved features of MadBub gene family detected by ConFeaX + TPR and kinase domain** – fasta file containing motifs and domains discovered by ConFeaX, including the TPR and kinase domain. Headers include four-letter species code, homolog type (MADBUB, BUB or MAD), domain name and position in the protein sequence (e.g. >HSAP_BUB||kinase/777-1038).

**Supplementary table I – species ID + full names table**  - This table can be used to look up species names and associated taxonomy for four letter codes used in supplementary sequences files and table II.

**Supplementary table II – Matrix of features in all MadBub orthologs** – Frequency matrix of conserved features reported by our ConFeaX pipeline for the MadBub gene family. Conserved functional motifs and domains are organized and colored in similar fashion as **figure 1, 2**. Names of duplicated species are in *italic*.

**Supplementary table III – primers used for molecular cloning**

**Supplementary figure 1 – Phylogenetic analysis of the MadBub gene family** (A) Maximum likelihood tree of the TPR region of 148 MadBub sequences. Blue circles indicate bootstrap support and the dashes red lined squares and asterisk (*) indicate which clades are associated with duplications. For further discussion see **supplementary discussion**. (B) Schematic representation of our reconciliation of the tree in panel A with the eukaryotic tree of life. This shows 16 independent duplication events throughout eukaryotic evolution. Arrows indicate duplications: orange (uncertain) and red (high confidence). Question marks point out clades in which the placement of the duplications can be debated, see for discussion **supplementary discussion**. Numbers in the tree correspond to duplications in specific taxa – containing the following species: {1}-mucorales; {2}-saccharomycetaceae; {3}-schizosaccharomycetes; {4}-pucciniomycetes: {5}-agaricomycetes (excluding early-branching species); {6}-vertebrates; {7}-teleost fish; {8}-nematodes; {9}-diptera (flies); {10}-albuginaceae (oomycete); {11}-ectocarpales (brow algae); {12}-aureococcus (harmful algae bloom); {13}-bryophytes (mosses); {14}-tracheophytes (vascular plants); {15}-magnoliaphytes (flowering plants); {16}-naegleria;

**Supplementary figure 2 – multiple sequence alignment of ABBA1-KEN2-ABBA2 cassette in all species used for this study** – Multiple sequence alignment of the ABBA1-KEN2-ABBA2 region in MADBUB and MAD paralogs of all sequences used in this study. The sequences of the alignment are grouped by relevant taxonomic levels. Colors are according to the Clustal scheme. Conservation is highlighted per group

**Supplementary procedures and discussion**

*Phylogenomic analyses*

Evolutionary relevant and divergent eukaryotic species were selected, in addition to our previously used set [1], based on their position relative to duplications and the inclusion of newly sequenced domains of the eukaryotic tree of life. Since the TPR domain is shared by all MadBub orthologs, we used HMMsearch [2] to capture and align the TPR domains in our dataset containing 152 genes in total. We selected only those columns of the multiple sequence alignment that had an occupancy of 80% or higher. RAxML [3] was used to perform phylogenetic analysis on in total 129 positions (2,1% gaps). A phylogenetic tree was estimated using the evolutionary model, selected by ProtTest [4] (LG + G). Parameters were set to be estimated, where possible. Confidence for the resulting maximum likelihood tree was assessed by a bootstrap analysis (1000 replicates). A schematic representation of the phylogenetic tree can be found in **figure 1b** and **supplementary figure 1b** based on our reconciliation of the maximum likelihood gene tree with the known species tree (**supplementary figure 1a**, for species names see **supplementary table I**), species taxonomy (Uniprot and newly published) and motif/domain content.

Similar to our previous findings, the maximum likelihood tree topology is fully inconsistent with a single duplication explaining all events in the MadBub gene family but neither are all independent duplications unambiguous and with maximal support present in the gene tree [1]. We could however still infer a manually reconciled tree based on the following pieces of information: First in some cases known whole genome duplications and their syntenic conservation provide unambiguous phylogenetic timing of duplication despite poorly supported or wrong topology in the gene tree. Second the presence of a single full length MadBub protein in the genomes of closely-related species that branch of just before a species where a BUB *and* a MAD protein are both present in the tree, even if they are not precisely inferred where they should be. There can be many reasons why such a short piece of sequence would hinder the correct inference of the evolutionary history of this gene family besides general lack of phylogenetic signal in so few amino acids. In some cases these inconsistencies are likely explained by the increased rate of evolution of one of the paralogs after duplication (mostly BUB). Low bootstrap support values furthermore signified the incorrect placement of a number of species, relative to the species tree (although most of the major eukaryotic supergroups were recovered). And thus in general if we would perform strict tree reconciliation on our full gene tree, unrealistic losses and or duplications had to be inferred. In any case, our current analysis strengthens our previous conclusion on recurrent independent duplications of an ancestral MadBub gene. We therefore focused on the duplication events in specific taxa and chose to guide our reconciliation by the motif/domain content if applicable. Relevant new evidence for duplication events is discussed in short below.

Our current analysis corroborated the 10 independent duplications previously described (vertebrates {#6,7}, diptera {#9}, nematodes {#8}, two in land plants {#13-15}, saccharomycetaceae {#2}, schizosaccharomyces {#3}, *L. bicolor* {#5, agaricomycetes}, *P. blakesleeanus* {#1, mucorales} and *N.* *gruberi* {#16} see **supplementary figure 1 a-b**) [1]. By the addition of sequences of recently sequenced genomes, we aimed to time the duplications more accurately and determine whether patterns of subfunctionalization were consistent between species after duplication. Unfortunately, the increased number of species did not aid to resolve the uncertain placement (see * in **supplementary figure 1a**) of duplications for schizosaccharomyces {#3} and the mucorales {#1}. We could not detect any excavate MadBub-like sequence, other then *N. gruberi*{#16}.

The presence of a single MadBub homolog in the early-branching (proto-) vertebrate *P. marinus* (lamprey) with an intact kinase domain, suggested that the duplication in vertebrates occurred after the divergence of lamprey. In our tree however, pmMadBub groups with the MAD paralog (RAxML 20) and it lacks the CMI motif (lost in vertebrate MAD, although present in *C. milli* MAD). Furthermore, the placement of lamprey relative to the major whole genome duplication in vertebrates is currently still under debate [5]. Consistent with the hypothesis of another whole genome duplication (3R) at the base of teleost fish, we find a third MadBub homolog in *D. rerio*. We could however not include this gene in our maximum likelihood analysis, since it lacked a N-terminal TPR domain. Strikingly, in most other teleost lineages this extra homolog has disappeared and only MAD A has remained (which lost its C-terminus), illustrating the potential fate of the MAD (BUBR1) paralog in other vertebrate lineages. Increased branch-lengths for the nematode and to a lesser extent diptera MadBub paralogs are reflected by the motif loss (CMI and KARD (only nematodes)) and degenerate nature of the motifs and domains in general (loss of KEN2 in nematodes), indicating extensive rewiring of SAC signaling after duplication in these taxa.

To elucidate the intriguing consecutive duplications in plants, we added MadBub orthologous sequences of early-branching plants (*K. flaccidum* and *Marchantia polymorpha*) and a number of flowering plants (*A. trichopoda*, *A. coerulea* and *Oryza sative japonica*). Reconciliation, taking into account the number of MadBub homologs, suggested a duplication in embryophytes (*P. patens* + *S. moelendorffii* – low support and unclear toplogy) followed by a duplication in of the BUB-like paralog in magnoliaphytes (RAxML 48). However, careful consideration of the motif and domain content of these sequences allowed for a more parsimonious explanation (from the domain/motif perspective): **(1)** independent duplication in *P. patens* (both paralogs have a GLEBS), **(2)** duplication in tracheophytes (loss of GLEBS, subfunctionalization into MAD (KEN1-ABBA1-KEN2-ABBA2-MadaM) and BUB (CMI-ABBA-CDII-kinase) and **(3)** duplication in magnoliaphytes (consecutive subfunctionalization of BUB into CDII+kinase (BUB A) and CMI+ABBA (BUB B)).

The four duplications we previously found in fungi, urged us to extend our search for duplications in newly sequenced species. We could more accurately time a previously found duplication in edible basidiomyceteous fungi {#5} of the agaricomycetes: early-branching lineages were found to only have a single MadBub homolog, containing all functional features of MAD and BUB (*E. glandulosa* and *R. solani*). Searching for additional MAD and BUB sequence we found a duplication in the basal basidiomycete fungi clade of the pucciniomycetes ({#4}, *M. larici-populina*, RAxML 22). (for phylogeny of basidiomycetes see [6].

Strikingly, we found evidence for three additional duplications in stramenopile species of the SAR super group (*A. laibachii* {#10}, *E. siliculosis* {#11} and *A. anophagefferens* {#12}). Although, the duplication of *A. anophagefferens* (RAxML 33) is not well supported and *E. siliculosis* MAD and BUB are grouped in different parts of the tree, the presence of an ancestral MadBub gene in a number of stramenopile lineage (diatoms and oomycetes), do not support a common ancestral duplication to have given rise to MAD and BUB in stramenopiles.

*Detailed discussion of BUB-related motifs and domains*

We observed three sub-clusters for the BUB-like paralog in our conserved feature correlation analysis (see **figure 1b** and **2a**):

**(1)** the CDII-kinase (*r*=0.94) cluster represents the most coherent BUB-associated cluster, having the highest anti-correlation score with the predominant MAD-associated features (-0.7<*r*<-0.64). Strikingly, the CDII and the kinase domain are occasionally lost in a number of SAR and archeaplastida species (*e.g.* stramenopiles, green –and red algae species, see **supplementary figure 1b**, **supplementary sequence file 1, supplementary table II**). Although, loss may reflect the common problem of gene prediction programs to correctly predict either amino –or carboxy terminal regions, the parallel nature of this events in different lineages advocates true loss, and would provide an opportunity to discover co-evolving features in other SAC-related protein such as members of the chromosomal passenger complex, which are localized through the catalytic activity of the BUB-related kinase domain [7].

**(2)** the clustering of CMI and ‘ABBA other’ motifs (*r*=0.56), although in close proximity in many species, is best illustrated by the secondary subfunctionalization of BUB A (TPR-CDII-kinase) and BUB B (TPR-[CMI-ABBA]^n^) in flowering plants (**figure 1b** {15}} following a duplication the ancestral BUB (TPR-CMI-CDII-kinase; vascular plants). In addition, the recurrent loss (*e.g.* in mucorales {1}, diptera {9} and the proto-vertebrate lamprey) and repeated nature in distinct lineages (archeaplastids, *G. theta* and *E. huxleyi*, see **figure 1b**, **supplementary figure 1b**), signify a distinct role of the CMI motif (+/- ABBA motif). Recent reports suggest that the region encompassing CMI is part of the elusive MAD1 kinetochore localization module, either through phospho-regulated interaction [8], through loading [9] or in an unknown manner through the RZZ complex [10]. Given the limited phylogenetic distribution of the RZZ complex to mainly opisthokonts species [11], the latter option does not seem the most likely function of the CMI motif. We favor the recently advocated template hypothesis[12], in which the strong correlation of CMI and close by ABBA motif signify a scaffold for MAD1 and CDC20, providing a platform for the formation of a c-MAD2-CDC20 dimer, primed to bind the MAD paralog.

**(3)** The KARD motif was mainly detected in opisthokonts, as part of both BUB (fungi and vertebrates) and MAD (diptera and vertebrates). The presence of this motif in the rhizarium *B. natans,* cryptophyte *G. theta* and the red algae *C. merolae* suggested an origin in LECA, but we deem it more likely that the KARD motif evolved de novo in these lineages or are false positive hits due to the degeneracy of the motif definition (**supplementary figure 1b**). The KARD - GLEBS association (*r*=0.43) illustrates the need for co-presence of these domains at the kinetochore. A similar pattern was observed for GLEBS and CMI (*r*=0.39, e*.g.* in MAD in *A. laibacchii* and *A. anophagefferens*) (**figure 1b**, **supplementary figure 1b**). Although crucial for proper kinetochore localization of MAD and BUB, through Bub3, the lineage –specific divergent sequences surrounding the GLEBS domain (N-terminal region of the defined feature by ConFeaX, **figure 1a**) indicate plastic evolution of the GLEBS-BUB3 kinetochore interaction, reminiscent of the widespread recurrent patterns of rapid repeat evolution of its major localizing phospho-motif, termed MELT [13]. In addition we observed the loss (vascular plants {13}, MAD: schizosaccharomyces {3}, basidiomycetes {4,5}) or occasional duplication of the GLEBS domain in animal lineages (diptera {9}, *Z. nevadensis*, *D. magna, N. vectensis*). A recently retracted paper of *Paganelli et al.* [14,15] reported the novel interaction of the MAD and BUB B paralogs in *A. thaliana* with MAP65-3, a member of an extensively diversified gene family in plants, which is orthologous to the human anti-parallel microtubule-crosslinking protein PRC. Interestingly, upon closer examination of this protein family, we discovered the de novo evolution of a GLEBS domain in a specific subset of the MAP65 paralogs (3 and 4). These findings suggest that MadBub kinetochore localization is regulated in a different/novel manner between species and maybe subject to forces favoring rapid evolution (positive selection).

**Supplementary References**

1. Suijkerbuijk, S. J. E. et al. 2012 The Vertebrate Mitotic Checkpoint Protein BUBR1 Is an Unusual Pseudokinase. *Dev. Cell* **22**, 1321–1329. (doi:10.1016/j.devcel.2012.03.009)

2. Finn, R. D., Clements, J. & Eddy, S. R. 2011 HMMER web server: Interactive sequence similarity searching. *Nucleic Acids Res.* **39**, W29-37. (doi:10.1093/nar/gkr367)

3. Stamatakis, A. 2014 RAxML version 8: a tool for phylogenetic analysis and post-analysis of large phylogenies. *Bioinformatics* **30**, 1312–3. (doi:10.1093/bioinformatics/btu033)

4. Darriba, D., Taboada, G. L., Doallo, R. & Posada, D. 2011 ProtTest 3: fast selection of best-fit models of protein evolution. *Bioinformatics* **27**, 1164–5. (doi:10.1093/bioinformatics/btr088)

5. Smith, J. J. et al. 2013 Sequencing of the sea lamprey (Petromyzon marinus) genome provides insights into vertebrate evolution. *Nat. Genet.* **45**, 415–421. (doi:10.1038/ng.2568)

6. Nagy, L. G. et al. 2016 Comparative Genomics of Early-Diverging Mushroom-Forming Fungi Provides Insights into the Origins of Lignocellulose Decay Capabilities. *Mol. Biol. Evol.* **33**, 959–70. (doi:10.1093/molbev/msv337)

7. Kawashima, S. a, Yamagishi, Y., Honda, T., Ishiguro, K. & Watanabe, Y. 2010 Phosphorylation of H2A by Bub1 prevents chromosomal instability through localizing shugoshin. *Science* **327**, 172–177. (doi:10.1126/science.1180189)

8. London, N. & Biggins, S. 2014 Mad1 kinetochore recruitment by Mps1-mediated phosphorylation of Bub1 signals the spindle checkpoint. *Genes Dev.* **28**, 140–152. (doi:10.1101/gad.233700.113)

9. Vleugel, M., Hoek, T. a., Tromer, E., Sliedrecht, T., Groenewold, V., Omerzu, M. & Kops, G. J. P. L. 2015 Dissecting the roles of human BUB1 in the spindle assembly checkpoint. *J. Cell Sci.* **128**, 2975–2982. (doi:10.1242/jcs.169821)

10. Zhang, G., Lischetti, T., Hayward, D. G. & Nilsson, J. 2015 Distinct domains in Bub1 localize RZZ and BubR1 to kinetochores to regulate the checkpoint. *Nat. Commun.* **6**, 7162. (doi:10.1038/ncomms8162)

11. Vleugel, M., Hoogendoorn, E., Snel, B. & Kops, G. J. P. L. 2012 Evolution and Function of the Mitotic Checkpoint. *Dev. Cell* **23**, 239–250. (doi:10.1016/j.devcel.2012.06.013)

12. Musacchio, A. 2015 The Molecular Biology of Spindle Assembly Checkpoint Signaling Dynamics. *Curr. Biol.* **25**, R1002–R1018. (doi:10.1016/j.cub.2015.08.051)

13. Tromer, E., Snel, B. & Kops, G. J. P. L. 2015 Widespread recurrent patterns of rapid repeat evolution in the kinetochore scaffold KNL1. *Genome Biol. Evol.* **7**, 2383–2393. (doi:10.1093/gbe/evv140)

14. 2016 Retraction. *New Phytol.* **212**, 1106–1106. (doi:10.1111/nph.14225)

15. Paganelli, L., Damiani, I., Govetto, B., Lecomte, P., Karpov, P. A., Abad, P., Chabout, M. & Favery, B. 2014 Three BUB1 and BUBR1 / MAD3-related spindle assembly checkpoint proteins are required for accurate mitosis in Arabidopsis. *New Phytol.* **205**, 202–215. (doi:10.1111/nph.13073)
