# Supplementary material for: Phylogenomics-guided discovery of a novel conserved cassette of short linear motifs in BubR1 essential for the spindle checkpoint
Source: Open Biol. 2016 Dec 21;6(12):160315. doi: 10.1098/rsob.160315 (PMC5204127; doi:10.1098/rsob.160315)
Supplement: Supplementary Figures 1-2 and tables I-III [file rsob160315supp2.pdf]

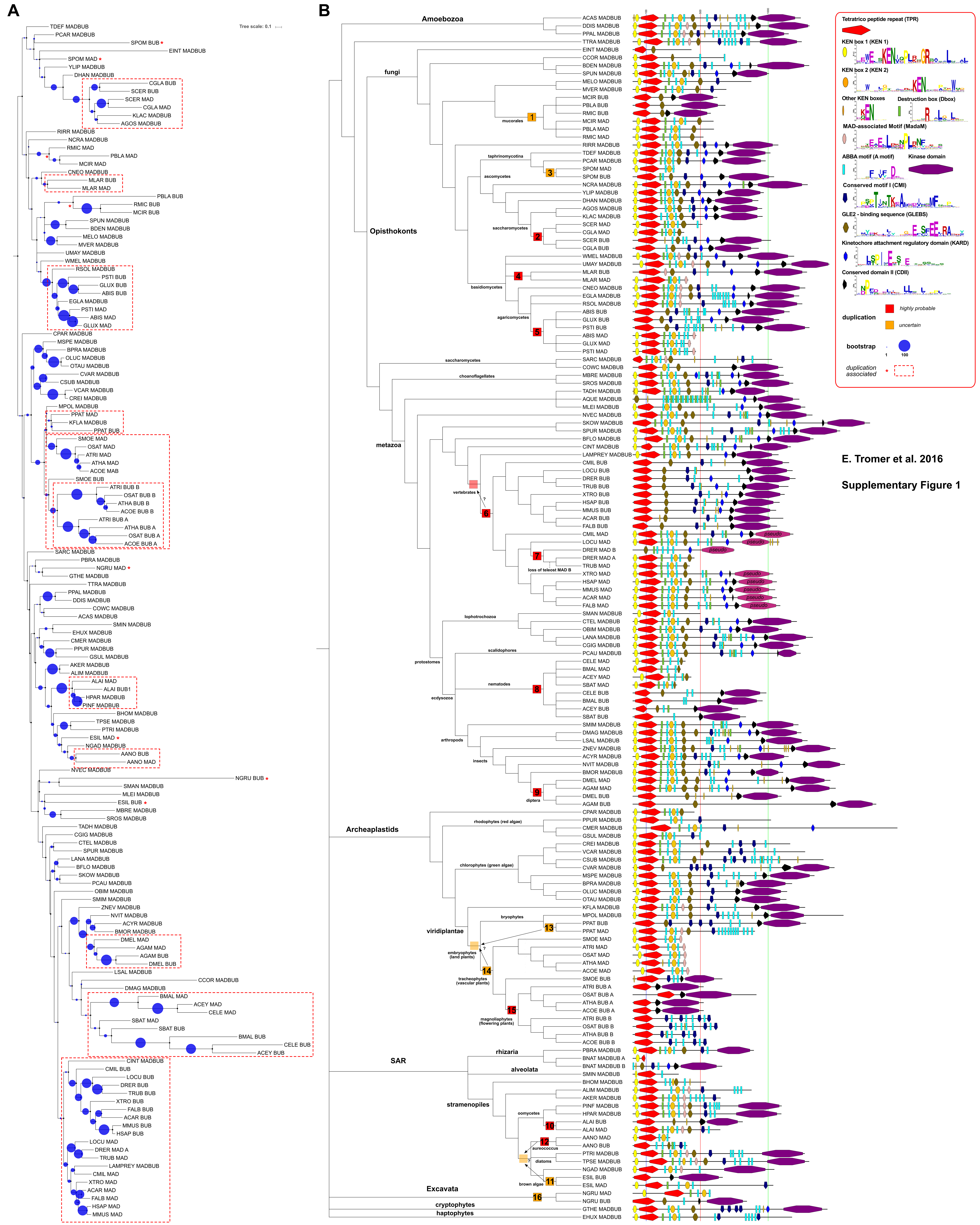

E. Tromer et al. 2016

Supplementary Figure 1

Supplementary file 2

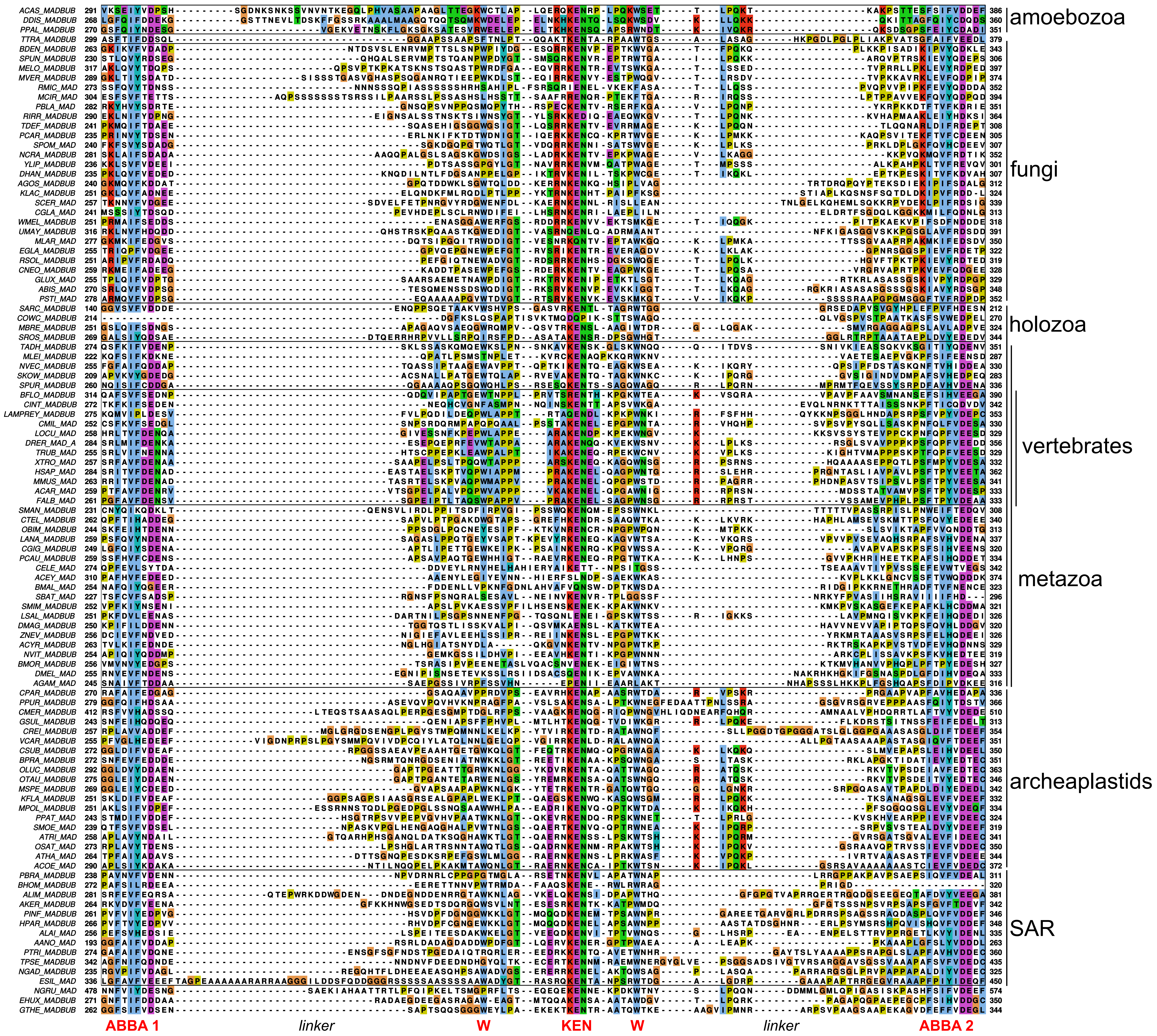

Supplementary table I

Taxonomy				Species ID	Species name
amoeba	longamoebia			ACAS	*Acanthamoeba castellanii*
	dictyostelia			DDIS	*Dictyostelium discoideum*
				PPAL	*Polysphondylium palidum*
apusomonadida				TTRA	*Thecamonas trahens*
opisthokonta	ichtyosporea			SARC	*Sphaeroforma arctica*
	filasterea			COWC	*Capsaspora owczarzaki*
	choanoflagellida			MBRE	*Monosiga brevicollis*
				SROS	*Salpingoeca rosetta*
	metazoa	chordata	vertebrata	LAMPREY	*Petromyzon marinus*
				CMIL	*Callorhinchus milii*
				LOCU	*Lepisosteus oculatus*
				DRER	*Danio rerio*
				TRUB	*Takifugu rubripes*
				FALB	*Ficedula albicollis*
				ACAR	*Anolis carolinensis*
				HSAP	*Homo sapiens*
				MMUS	*Mus musculus*
				XTRO	*Xenopus tropicalis*
			cephalochordata	BFLO	*Branchiostoma floridae*
			tunicata	CINT	*Ciona intestinales*
		echinodermata		SPUR	*Strongylocentrotus purpuratus*
		hemichordata		SKOW	*Saccoglossus kowalevskii*
		annelida		CTEL	*Capitella teleta*
		brachiopoda		LANA	*Lingula anatina*
		mollusca		CGIG	*Crassostrea gigas*
				OBIM	*Octopus bimaculoides*
		platyhelmithes		SMAN	*Schistosoma mansoni*
		vinctiplicata		PCAU	*Priapulus caudatus*
		nematoda	rhabditida	CELE	*Caenorhabditis elegans*
				ACEY	*Ancylostoma ceylanicum*
			spirurida	BMAL	*Brugia maly*
			ascaridida	SBAT	*Soboliphyme baturini*
		arthropoda	insecta	DMEL	*Drosophila melanogaster*
				AGAM	*Anopheles gambiae*
				BMOR	*Bombyx mori*
				NVIT	*Nasonia vitripennis*
				ACYR	*Acyrthrosiphon pisum*
				ZNEV	*Zootermopsis nevadensis*
			crustacea	LSAL	*Lepeophtheirus salmonis*
				DMAG	*Daphnia magna*
			arachnida	SMIM	*Stegodyphus mimosarum*
		cnidaria		NVEC	*Nematostella vectensis*
		placozoa		TADH	*Trichoplax adhaerens*
		ctenophora		MLEI	*Mnemiopsis leidiyi*
		porifera		AQUE	*Amphimedon queenslandica*
	fungi	microsporidia		EINT	*Encephalitozoon intestinalis*
		chytridiomycota		BDEN	*Batrachochytrium dendrobatidis*
				SPUN	*Spizellomyces punctatus*
		mucorales		RMIC	*Rhizopus microsporus*
				MCIR	*Mucor circinelloides*
				PBLA	*Phycomyces blakesleeanus*
		mortierellaceae		MVER	*Mortierella verticillata*
				MELO	*Mortierella elongata*
		glomeromycota		RIRR	*Rhizophagus irregularis*
		entomophtorales		CCOR	*Conidiobolus coronatus*
		basidiomycota	ustilaginomycetes	UMAY	*Ustilago maydis*
			wallemiomycetes	WMEL	*Wallemia mellicola*
			pucciniomycetes	MLAR	*Melampsora larici-populina*
			tremellomycetes	CNEO	*Cryptococcus neoformans*
			agaricomycetes	RSOL	*Rhizoctonia solani*
				EGLA	*Exidia glandulosa*
				PSTI	*Punctularia strigosozonata*
				ABIS	*agaricus bisporus*
				GLUX	*Gymnopus luxurians*
		ascomycota	taphrinomycotina	PCAR	*Pneumocystis carinii*
				TDEF	*Taphrina deformans*
				SPOM	*Schizosaccharomyces pombe*
			pezizomycetes	NCRA	*Neurospora crassa*
			saccharomycetes	YLIP	*Yarrowia lipolytica*
				DHAN	*Debaryomyces hansenii*
				KLAC	*Kluyveromyces lactis*
				AGOS	*Ashbya gossipii*
				CGLA	*Candida glabrata*
				SCER	*Saccharomyces cerevisiae*
archeaplastida	glaucophyta			CPAR	*Cyanophora paradoxa*
	rhodophyta		cyanidales	GSUL	*Galdieria sulphuraria*
				CMER	*Cyanidioschyzon merolae*
			porphyridiales	PPUR	*Porphyridium purpureum*
	viridiplantae	chlorophyta	mamiellophyceae	BPRA	*Bathycoccus prasinos*
				MSPE	*Micromonas species*
				OLUC	*Ostreococcus lucimarinus*
				OTAU	*Ostreococcus tauri*
			chlorophyceae	CREI	*Chlamydomonas reinhardtii*
				VCAR	*Volvox carteri*
			trebouxiophyceae	CVAR	*Chlorella variabilis*
				CSUB	*Coccomyxa subellipsoidea*
		klebsormidiophyta		KFLA	*Klebsormidium flaccidum*
		embryophyta	marchantiophyta	MPOL	*Marchantia polymorpha*
			bryophyta	PPAT	*Physcomitrella patens*
			lycopodiophyta	SMOE	*Selaginella moellendorffii*
			magnoliaphyta	ATRI	*Amborella trichopoda*
				OSAT	*Oryza sativa japonica*
				ATHA	*Arabidopsis thaliana*
				ACOE	*Aquilegia coerulea*
excavata	heterolobosoea			NGRU	*Naegleria gruberi*
SAR	stramenopiles	blastocystis		BHOM	*Blastocystis hominis*
		labyrinthulomycetes		AKER	*Aplanochytrium kerguelense*
				ALIM	*Aurantiochytrium limacinum*
		oomycetes		ALAI	*Albugo laibachii Nc*
				PINF	*Phythophthora infestans*
				HPAR	*Hyaloperonospora parasitica*
		eustigmatales		NGAD	*Nannochloropsis gaditana*
		pelagophyceae		AANO	*Aureococcus anophagefferens*
		phaephyceae		ESIL	*Ectocarpus siliculosus*
		diatomea		PTRI	*Phaeodactylum tricornutum*
				TPSE	*Thalassiosira pseudonana*
	rhizaria	plasmodiophoromycetes		PBRA	*Plasmodiophora brassicae*
		cercozoa		BNAT	*Bigelowiella natans*
	alveolata	chlorarachniophyta		SMIN	*Symbiodinium minutum*
haptophyta				EHUX	*Emiliania huxleyi*
cryptophyta				GTHE	*Guillardia theta*

Supplementary table II

eukaryotic supergroup	Species ID	ID|type	TPR	MAD-like								BUB-like					
				KEN1	ABBA1	KEN2	ABBA2	Dbox	MadaM	GLEBS		CMI	ABBA other	KARD	KEN other	CDII	kinase
amoeba	ACAS	ACAS_MADBUB	1	1	1	1	1	1		1		1	4		3	1	1
	DDIS	DDIS_MADBUB	1	1	1	1	1	1	1	1		1	3		2	1	1
	PPAL	PPAL_MADBUB	1	1	1	1	1	1	1	1		1	6		1	1	1
unknown	TTRA	TTRA_MADBUB	1	1	1	1	1	1	1	1		2	6				1
opisthokonta	SARC	SARC_MADBUB	1		1	1	1	1	1	1		1	2		2		
	COWC	COWC_MADBUB	1			1	1			1						1	1
	MBRE	MBRE_MADBUB	1	1	1	1	1	2		1		1	3	2		1	1
	SROS	SROS_MADBUB	1	1	1	1	1	2		1		1	1	1	1	1	1
	LAMPREY	LAMPREY_MADBUB	1	1	1	1	1	1		1				1		1	1
	CMIL	CMIL_BUB	1							1		1	1		1	1	1
		CMIL_MAD	1	1	1	1	1	3		1		1	1	1		1	1
	LOCU	LOCU_BUB	1							1		1	1		1	1	1
		LOCU_MAD	1	1	1	1	1	2		1			2	1	3		1
	DRER	DRER_BUB	1							1		1	2	1		1	1
		DRER_MAD_A	1	1	1	1	1	1		1							
		DRER_MAD_B						1		1			4	1			1
	TRUB	TRUB_BUB	1							1		1	2	1	1	1	1
		TRUB_MAD	1	1	1	1	1	1		1							
	FALB	FALB_BUB	1							1		1	1		2	1	1
		FALB_MAD	1	1	1	1	1	2		1			2	1		1	1
	ACAR	ACAR_BUB	1							1		1	1		1	1	1
		ACAR_MAD	1	1	1	1	1	1		1			1	1	1	1	1
	HSAP	HSAP_BUB	1							1		1	2	1	2	1	1
		HSAP_MAD	1	1	1	1	1	2		1			1	1		1	1
	MMUS	MMUS_BUB	1							1		1	1	1	3	1	1
		MMUS_MAD	1	1	1	1	1	2		1			1	1		1	1
	XTRO	XTRO_BUB	1							1		1	1	1	1	1	1
		XTRO_MAD	1	1	1	1	1	2		1			1	1		1	1
	BFLO	BFLO_MADBUB	1	1	1	1	1	2		1		1	1	1	2	1	1
	CINT	CINT_MADBUB	1	1	1	1	1	2		1		1	4	1		1	1
	SPUR	SPUR_MADBUB	1	1	1	1	1	2		1		1	7	1	1	1	1
	SKOW	SKOW_MADBUB	1		1	1	1	1		1		1	1	1	2	1	1
	CTEL	CTEL_MADBUB	1	1	1	1	1	1		1		1	2	1	1	1	1
	LANA	LANA_MADBUB	1	1	1	1	1	3		1		1	4	1	1	1	1
	CGIG	CGIG_MADBUB	1	1	1	1	1	1		1		1	2	1	1	1	1
	OBIM	OBIM_MADBUB	1	1	1	1	1	1		1		1	1	1		1	1
	SMAN	SMAN_MADBUB	1	1	1	1	1										
	PCAU	PCAU_MADBUB	1	1	1	1	1	2		1		1	3	1	1	1	1
	CELE	CELE_BUB	1							1			2			1	1
		CELE_MAD	1	1	1		1	1		1							
	ACEY	ACEY_BUB	1					1		1			1			1	1
		ACEY_MAD	1	1	1		1	1		1					1		
	BMAL	BMAL_BUB	1										2			1	1
		BMAL_MAD	1	1	1		1	1		1							
	SBAT	SBAT_BUB	1										2			1	1
		SBAT_MAD	1	1	1	1	1	1		1							
	DMEL	DMEL_BUB	1							1					3	1	1
		DMEL_MAD	1	1	1	1	1	1		2			3	1	2	1	1
	AGAM	AGAM_BUB	1							1						1	1
		AGAM_MAD	1	1	1	1	1	1		2			2	1	2	1	1
	BMOR	BMOR_MADBUB	1	1	1	1	1	1		1		1	1	1	2	1	1
	NVIT	NVIT_MADBUB	1	1	1	1	1	1		1		1	2	1	2	1	1
	ACYR	ACYR_MADBUB	1	1	1	1	1	1		1		1	5	1		1	1
	ZNEV	ZNEV_MADBUB	1	1	1	1	1	3		2		1	2	1	7	1	1
	LSAL	LSAL_MADBUB	1	1	1	1	1	1		1			3	1	1	1	1
	DMAG	DMAG_MADBUB	1	1	1	1	1	3		2		1	2	1	2	1	1
	SMIM	SMIM_MADBUB	1	1	1	1	1	2		1			3	1		1	1
	NVEC	NVEC_MADBUB	1	1	1	1	1	2		2		1	3	1	1	1	1
	TADH	TADH_MADBUB	1	1	1	1	1	2		1			2	1	2	1	1
	MLEI	MLEI_MADBUB	1	1	1	1	1			1			3	1		1	1
	AQUE	AQUE_MADBUB						11		1			10	1	1	1	1
	EINT	EINT_MADBUB	1							1							
	BDEN	BDEN_MADBUB	1	1	1	1	1	1		1		1	2	1		1	1
	SPUN	SPUN_MADBUB	1	1	1	1	1	1		1		1	1	1	1	1	1
	RMIC	RMIC_BUB	1							1						1	1
		RMIC_MAD	1	1	1	1	1	1		1							
	MCIR	MCIR_BUB	1							1						1	1
		MCIR_MAD	1	1	1	1	1	1		1							
	PBLA	PBLA_BUB	1							1							1
		PBLA_MAD	1	1	1	1	1	1		1							
	MVER	MVER_MADBUB	1	1	1	1	1	1		2		1					
	MELO	MELO_MADBUB	1	1	1	1	1	1		1		1					
	RIRR	RIRR_MADBUB	1	1	1	1	1	1		1		1		1		1	1
	CCOR	CCOR_MADBUB	1	1						1			2				
	UMAY	UMAY_MADBUB	1	1	1	1	1	1	1	1		1		1		1	1
	WMEL	WMEL_MADBUB	1	1	1	1	1	1	1	1		1	1	1		1	1
	MLAR	MLAR_BUB	1						1	1		1	1	1		1	1
		MLAR_MAD	1	1	1	1	1	1	1								
	CNEO	CNEO_MADBUB	1	1	1	1	1	1	1	1		1	2	1		1	1
	RSOL	RSOL_MADBUB	1	1	1	1	1	1	1			1	5	1		1	1
	EGLA	EGLA_MADBUB	1	1	1	1	1	1	1	1		1	7	1		1	1
	PSTI	PSTI_BUB	1					1		1		1	7	1	1	1	1
		PSTI_MAD	1	1	1	1	1	1	1				2				
	ABIS	ABIS_BUB	1					1		1		1	5	1		1	1
		ABIS_MAD	1	1	1	1	1	1	1				2				
	GLUX	GLUX_BUB	1							1		1	4	1		1	1
		GLUX_MAD	1	1	1	1	1	1	1				2				
	PCAR	PCAR_MADBUB	1	1	1	1	1	1		1		1		1		1	1
	TDEF	TDEF_MADBUB	1	1	1	1	1	1		1		1	1	1		1	1
	SPOM	SPOM_BUB	1				1	1		1		1		1		1	1
		SPOM_MAD	1	1	1	1	1										
	NCRA	NCRA_MADBUB	1	1	1	1	1	2		1		1		1		1	1
	YLIP	YLIP_MADBUB	1	1	1	1	1	1		1		1		1		1	1
	DHAN	DHAN_MADBUB	1	1	1	1	1	1		1		1		1		1	1
	KLAC	KLAC_MADBUB	1	1	1	1	1			1		1	1	1		1	1
	AGOS	AGOS_MADBUB	1	1	1	1	1			1		1	1	1		1	1
	CGLA	CGLA_BUB	1							1		1	1	1		1	1
		CGLA_MAD	1	1	1	1	1	1		1							
	SCER	SCER_BUB	1							1		1	3	1	1	1	1
		SCER_MAD	1	1	1	1	1	1		1			1				
archeaplastida	CPAR	CPAR_MADBUB	1	1	1	1	1	1		1							
	GSUL	GSUL_MADBUB	1	1	1	1	1	1				1	1				
	CMER	CMER_MADBUB	1	1	1	1	1	1				1		1	1		
	PPUR	PPUR_MADBUB	1	1	1	1	1					1					
	BPRA	BPRA_MADBUB	1	1	1	1	1			1		1	1		2	1	1
	MSPE	MSPE_MADBUB	1	1	1	1	1			1		1	4			1	1
	OLUC	OLUC_MADBUB	1	1	1	1	1			1		1				1	1
	OTAU	OTAU_MADBUB	1	1	1	1	1			1		1				1	1
	CREI	CREI_MADBUB	1	1	1	1	1	1		1		2	2				
	VCAR	VCAR_MADBUB	1	1	1	1	1	1		1		2	2				
	CVAR	CVAR_MADBUB	1	1						1		4	2		1	1	1
	CSUB	CSUB_MADBUB	1	1	1	1	1	1		1		3	8		3		
	KFLA	KFLA_MADBUB	1	1	1	1	1	1	1	1		1	2			1	1
	MPOL	MPOL_MADBUB	1	1	1	1	1			1		1	4		1	1	1
	PPAT	PPAT_BUB	1							1		4	2			1	1
		PPAT_MAD	1	1	1	1	1		1	1		1	11				
	SMOE	SMOE_BUB	1									1	2		1	1	1
		SMOE_MAD	1	1	1	1	1		1								
	ATRI	ATRI_BUB_A	1													1	1
		ATRI_BUB_B	1									4	4				
		ATRI_MAD	1	1	1	1	1		1								
	OSAT	OSAT_BUB_A	1													1	1
		OSAT_BUB_B	1									3	5		1		
		OSAT_MAD	1	1	1	1	1		1								
	ATHA	ATHA_BUB_A	1													1	1
		ATHA_BUB_B	1									3	3		1		
		ATHA_MAD	1	1	1	1	1		1						1		
	ACOE	ACOE_BUB_A	1													1	1
		ACOE_BUB_B	1									3	3				
		ACOE_MAD	1	1	1	1	1		1								
excavata	NGRU	NGRU_BUB	1							1			1			1	1
		NGRU_MAD	1	1	1	1	1	1									
SAR	BHOM	BHOM_MADBUB	1	1	1	1				1			2		1		
	AKER	AKER_MADBUB	1	1	1	1	1	1	1			1	4				
	ALIM	ALIM_MADBUB	1	1	1	1	1		1			1	3				
	ALAI	ALAI_BUB	1							1					1	1	1
		ALAI_MAD	1	1	1	1	1	1	1	1		1	1				
	PINF	PINF_MADBUB	1	1	1	1	1	1	1	1		1	4				1
	HPAR	HPAR_MADBUB	1	1	1	1	1	1	1	1		1	2				1
	NGAD	NGAD_MADBUB	1	1	1	1	1		1				1				1
	AANO	AANO_BUB	1					1		1		1	4				
		AANO_MAD	1	1	1	1	1										
	ESIL	ESIL_BUB	1													1	1
		ESIL_MAD	1	1	1	1	1	1				1					
	PTRI	PTRI_MADBUB	1	1	1	1	1	1	1	1		1	4				1
	TPSE	TPSE_MADBUB	1	1	1	1	1	1	1	1		1	4				1
	PBRA	PBRA_MADBUB	1	1	1	1	1	1		1		1	1				1
	BNAT	BNAT_MADBUB_A	1	1													
		BNAT_MADBUB_B								1		1	2	1	1	1	1
	SMIN	SMIN_MADBUB	1	1	1												
unknown	EHUX	EHUX_MADBUB	1	1	1	1	1	1		1		2	5				
	GTHE	GTHE_MADBUB	1	1	1	1	1			1		4	2	1	1		1

Supplementary table III

mutant	5'-3' sequence	orientation
dABBA1	CCATTTCCTCAACAGATGCAAAATAATAGTGCTGATGAGGCTTCTACAGCAGAGTTGTCT	FW
dABBA1	AGACAACTCTGCTGTAGAAGCCTCATCAGCACTATTATTTTGCATCTGTTGAGGAAATGG	RV
dABBA2	TCACTGATAGCTGTACCCGCTGTGCTTCCCACTGCACAACAGCCAGTTATGACACCATGT	FW
dABBA2	ACATGGTGTCATAACTGGCTGTTGTGCAGTGGGAAGCACAGCGGGTACAGCTATCAGTGA	RV
W1A	CAGTCCAGCCAgcGATAGCACCCCCC	FW
W1A	GGGGGGTGCTATCgcTGGCTGGACTG	RV
W2A	GCAAGCAGGCCCTGCGAACACAGGCAGG	FW
W2A	CCTGCCTGTGTTCGCAGGGCCTGCTTGC	RV
KEN1-AAA	GATGAATGGGAACTGAGTGCAGCAGCTGTACAACCTTTAAGGCAAGG	FW
KEN1-AAA	CCTTGCCTTAAAGGTTGTACAGCTGCTGCACTCAGTTCCCATTCATC	RV
KEN2-AAA	CCATGCCCAGGGCAGCGGCTAGCGAGCTGCAAGC	FW
KEN2-AAA	GCTTGCAGCTCGCTAGCCGCTGCCCTGGGCATGG	RV
dDBOX	GAGTCTTCTGTACCACAAAAGAGCAAAGGGAAAAAGACAGC	FW
dDBOX	GCTGTCTTTTTCCCTTTGCTCTTTTGTGGTACAGAAGACTC	RV
dABBA3	GGTCCCAGTGTACCTTTTCTTCTTTCAGAAAAGAAG	FW
dABBA3	CTTCTTTTCTGAAAGAAGAAAAGGTACACTGGGACC	RV
